# Photoinduced synthesis of unsymmetrical diaryl selenides from triarylbismuthines and diaryl diselenides

**DOI:** 10.3762/bjoc.9.127

**Published:** 2013-06-13

**Authors:** Yohsuke Kobiki, Shin-ichi Kawaguchi, Takashi Ohe, Akiya Ogawa

**Affiliations:** 1 Department of Applied Chemistry, Graduate School of Engineering, Osaka Prefecture University, 1-1 Gakuen-cho, Nakaku, Sakai, Osaka 599-8531, Japan

**Keywords:** arylation, unsymmetrical diaryl selenide, free radical, organobismuth, photoinduced reaction

## Abstract

A novel method of photoinduced synthesis of unsymmetrical diaryl selenides from triarylbismuthines and diaryl diselenides has been developed. Although the arylation reactions with triarylbismuthines are usually catalyzed by transition-metal complexes, the present arylation of diaryl diselenides with triarylbismuthines proceeds upon photoirradiation in the absence of transition-metal catalysts. A variety of unsymmetrical diaryl selenides can be conveniently prepared by using this arylation method.

## Introduction

**Scheme 1:**
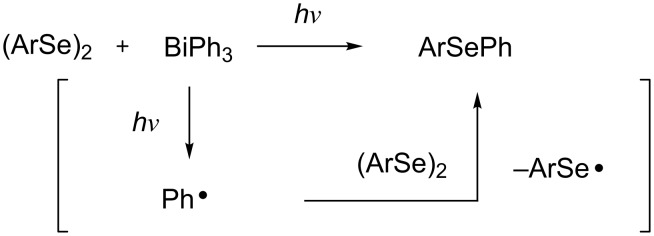
Photoinduced radical reaction of diaryl diselenide with triphenylbismuthine.

A number of organoselenium compounds are known to be biologically active [1–4]. In particular, diaryl selenides are known to have antioxidative effects [5]. Therefore, many studies on the synthetic methods for unsymmetrical diaryl selenides have recently been reported [6–32]. Most of these methods use coupling reactions catalyzed by transition-metal complexes. To avoid the contamination of product selenides with transition-metals, the development of synthetic methods for unsymmetrical diaryl selenides in the absence of transition-metal catalysts is desirable. On the other hand, triarylbismuthines are gaining interest as useful arylation reagents, because organobismuth compounds are nontoxic and have excellent reactivity, which has led to several applications in organic synthesis [33]. Therefore, numerous transition-metal-catalyzed coupling reactions with organobismuth compounds have been reported [34–53]. Although triphenylbismuthine can generate a phenyl radical [33,54] in the absence of a radical initiator simply by photoirradiation, few arylation reactions using this mechanism have been reported [55–56]. We presume that a phenyl radical generated from triphenylbismuthine can be captured by organic diselenides, which have a high carbon-radical-capturing ability [57–64] and as a result, diaryl selenide will be generated (Scheme 1). In 1999, Barton and co-workers reported that diaryl selenide was obtained by the reaction of triarylbismuthine with diphenyl diselenide under heating at high temperature (140 °C) [65], but the photoinduced reaction was not investigated. In this letter, we will report the radical reaction of diaryl diselenides with triarylbismuthines from the viewpoint of a photoinduced reaction in the synthesis of unsymmetrical diaryl selenides.

## Results and Discussion

First, we investigated the photoinduced reaction of diphenyl diselenide with triphenylbismuthine. Diphenyl diselenide (**1a**, 0.1 mmol) and triphenylbismuthine (**2a**, 0.5 mmol) were placed in a Pyrex test tube (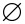 = 9 mm) with CHCl_3_ (4 mL), and the mixture was irradiated by a xenon lamp for 5 h at room temperature. As a result, 0.042 mmol (21% yield based on the amount of selenium atoms) of diphenyl selenide (**3aa**) was obtained after the isolation by silica gel chromatography (the yield was determined by HPLC). Next, optimization of the reaction conditions was investigated as shown in Table 1. Irradiation by a tungsten lamp instead of a xenon lamp did not induce the desired arylation reaction (Table 1, entry 2), and in the dark, the reaction did not proceed at all (Table 1, entry 3). When 2,2′-azobis(isobutyronitrile) (AIBN) was used as a radical initiator, the desired reaction proceeded ineffectively (Table 1, entry 4). Among several solvents, such as benzene, DMSO and CH_3_CN, the use of CH_3_CN improved the yield of **3aa** (Table 1, entries 5–7). Although the solubility of **2a** is different depending on the solvent, the yield of **3aa** is not correlated with the solubility of **2a**. It may be more important to choose a solvent that does not react with the generated aryl radical. Moreover, a lower amount of solvent and the utilization of a quartz test tube (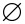 = 9 mm) contributed to the increase in the yield of **3aa** (Table 1, entries 8 and 9).

**Table 1:** Reaction of diphenyl diselenide with triphenylbismuthine under different conditions.

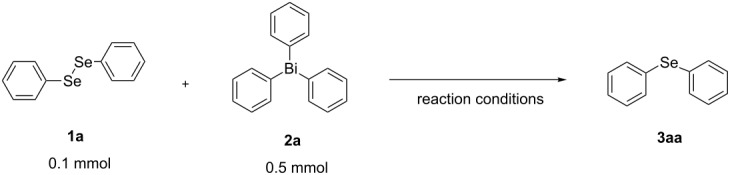		

entry	reaction conditions	yield of **3aa**^a^

1	CHCl_3_ (4 mL), xenon lamp, Pyrex test tube, 5 h	0.042 mmol, 21%
2	CHCl_3_ (4 mL), tungsten lamp, Pyrex test tube, 5 h	0.004 mmol, 2%
3	CHCl_3_ (4 mL), dark, 24 h	0%
4	C_6_H_6_ (5 mL), AIBN (1.5 mmol), 80 °C, two-necked flask, 8 h	0.012 mmol, 6%
5	C_6_H_6_ (4 mL), xenon lamp, Pyrex test tube, 5 h	0.102 mmol, 51%
6	DMSO (4 mL), xenon lamp, Pyrex test tube, 5 h	0.034 mmol, 17%
7	CH_3_CN (4 mL), xenon lamp, Pyrex test tube, 5 h	0.114 mmol, 57%
8	CH_3_CN (2 mL), xenon lamp, Pyrex test tube, 5 h	0.126 mmol, 63%
9	CH_3_CN (2 mL), xenon lamp, quartz test tube, 5 h	0.138 mmol, 69%

^a^The yields were determined by HPLC.

Next, we investigated the scope of the synthesis of unsymmetrical diaryl selenides by using different diaryl diselenides and triarylbismuthines (Table 2). The employed diaryl diselenides were diphenyl diselenide (**1a**), bis(4-fluorophenyl) diselenide (**1b**), bis(4-(trifluoromethyl)phenyl) diselenide (**1c**), bis(1-naphthyl) diselenide (**1d**), and bis(2-naphthyl) diselenide (**1e**). The used triarylbismuthines were triphenylbismuthine (**2a**), tris(4-methylphenyl)bismuthine (**2b**), tris(4-chlorophenyl)bismuthine (**2c**), and tris(4-fluorophenyl)bismuthine (**2d**). A number of combinations of **1** and **2** were examined and as a result, unsymmetrical diaryl selenides **3** were obtained in moderate to high yields (45–86%) in every case (Table 2, entries 1–10) after the isolation by preparative TLC on silica gel. The chemical shifts of ^77^Se NMR spectra of diaryl selenides **3** are also shown in Table 2, because ^77^Se NMR is a tool well suited to identify diorganyl monoselenides.

**Table 2:** Syntheses of unsymmetrical diaryl selenides.

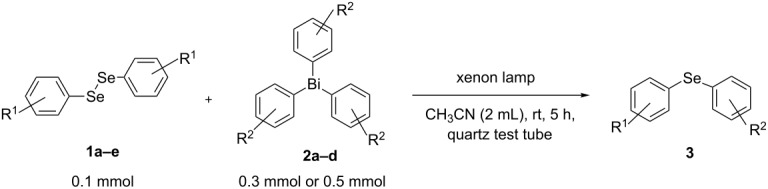					

entry	(ArSe)_2_ **1**	Ar’_3_Bi **2**	product **3** (ArSeAr’)	^77^Se NMR, δ ppm	yield^a^

1^b^	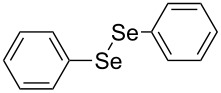 **1a**	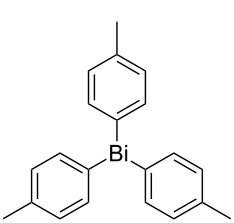 **2b**	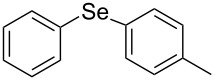 **3ab**	407	65%
2^b^	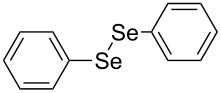 **1a**	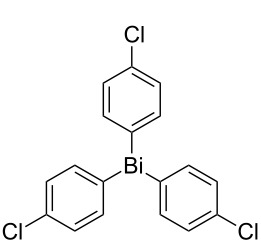 **2c**	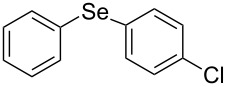 **3ac**	416	45%
3^c^	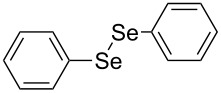 **1a**	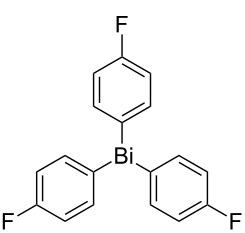 **2d**	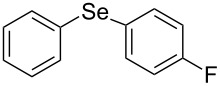 **3ad**	411	66%
4^b^	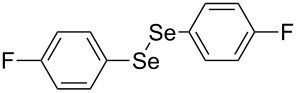 **1b**	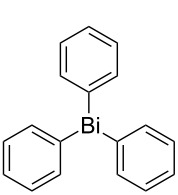 **2a**	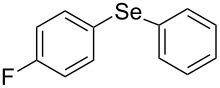 **3ba**	411	57%
5^b^	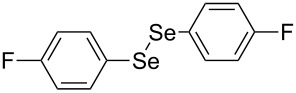 **1b**	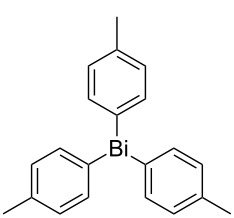 **2b**	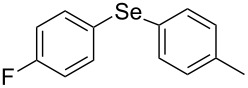 **3bb**	404	86%
6^b^	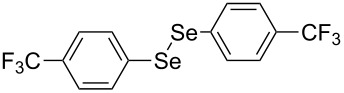 **1c**	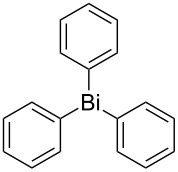 **2a**	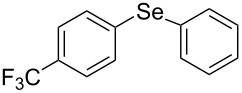 **3ca**	427	67%
7^b^	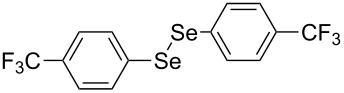 **1c**	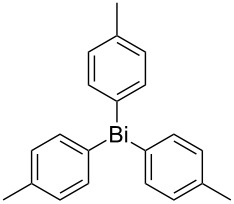 **2b**	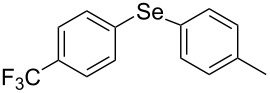 **3cb**	418	51%
8^c^	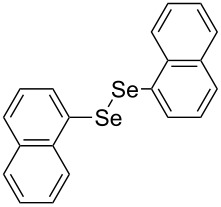 **1d**	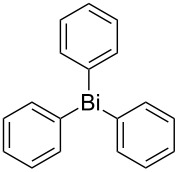 **2a**	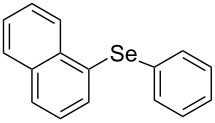 **3da**	355	51%
9^c^	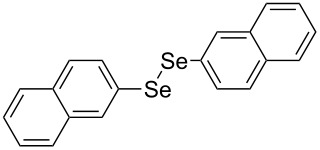 **1e**	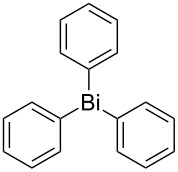 **2a**	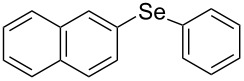 **3ea**	418	71%
10^c^	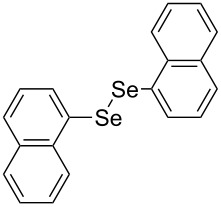 **1d**	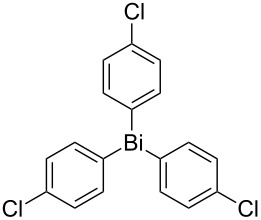 **2c**	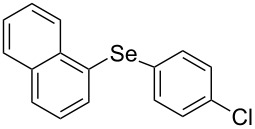 **3dc**	354	57%

^a^The yields were determined after isolation. ^b^0.5 mmol of triarylbismuthine was used. ^c^0.3 mmol of triarylbismuthine was used.

To get information about the reaction pathway of this arylation, we first investigated the arylation of diphenyl diselenide by varying the **1a**/**2a** molar ratio (Table 3). When excess amounts of either starting substrate were employed, the yields of **3aa** increased (Table 3, entries 1, 2 and 5).

**Table 3:** The yield of diphenyl selenide **3aa** upon changing the ratio **1a**/**2a**.

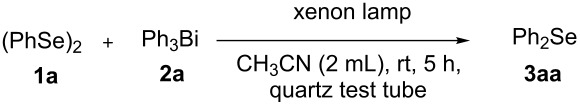			

entry	amount of **1a**	amount of **2a**	yield of **3aa**^a^

1	0.1 mmol	0.5 mmol	69% 0.138 mmol
2	0.1 mmol	0.3 mmol	69% 0.138 mmol
3	0.1 mmol	0.1 mmol	59% 0.118 mmol
4	0.1 mmol	0.067 mmol (2/3 equiv)	57% 0.118 mmol
5	0.2 mmol	0.1 mmol	88%^b^ 0.263 mmol

^a^The yields were determined by HPLC based on the amount of **1a**. ^b^The yield was calculated based on the amount of **2a**.

**Scheme 2:**
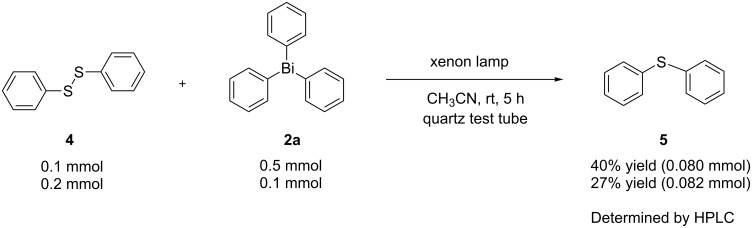
Photoinduced reaction of diphenyl disulfide with triphenylbismuthine.

In the case of the reaction of triphenylbismuthine with diphenyl disulfide (**4**) instead of diphenyl diselenide, diphenyl sulfide **5** was obtained in lower yield with unidentified byproducts, unlike in the case of diphenyl selenide **3aa** (Scheme 2).

Additionally, air is entrained in the reaction system, since a test tube with a septum was used in which a needle was inserted. When the reaction of diaryl diselenide with triarylbismuthine was conducted with a strictly sealed tube in Ar atmosphere, a bismuth mirror was observed and the yield of **3aa** decreased. We assume that the reaction proceeds with bismuth residue getting oxidized.

**Scheme 3:**
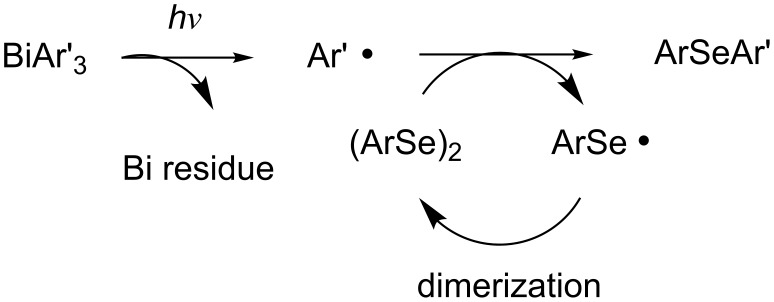
A plausible reaction pathway for the photoinduced reaction of diaryl diselenide with triarylbismuthine.

A plausible reaction pathway for the photoinduced reaction of diaryl diselenide with triarylbismuthine is shown in Scheme 3. First, an aryl radical is generated from triarylbismuthine by near-UV light irradiation [33,54–55]. The generated aryl radical is captured by diaryl diselenide to produce diaryl selenide and a seleno radical. The seleno radical may dimerize to re-form diselenide. Diphenyl diselenide has its absorption maximum (λ_max_) at 340 nm (ε = 10^3^) [66] and accordingly, the seleno radical could be produced by the irradiation with a tungsten lamp. However, the irradiation by a tungsten lamp instead of a xenon lamp did not result in the desired reaction (Table 1, entry 3). This fact strongly suggests that the formation of a phenylseleno radical is not important for the formation of diphenyl selenide. Conceivably, when the reaction proceeds, a phenyl radical may be formed directly from triphenylbismuthine upon photoirradiation. Moreover, the use of an excess amount of **1**, which has a relatively high carbon-radical-capturing ability, increased the yield of **3**, and the use of diphenyl disulfide (**4**), which has a lower carbon-radical-capturing ability than diselenide, decreased the yield of **5**. (The exact capturing abilities of diselenide and disulfide toward the phenyl radical are not known, but they have been reported toward vinyl radicals, where diselenide has a higher capturing ability than disulfide: *k*_Se_/*k*_S_ = 160 [57–59].) These facts also support that the reaction starts from the generation of an aryl radical. On the other hand, a pale yellow solid, insoluble in organic solvents, was obtained as a byproduct after the reaction. We assume that this solid is a bismuth residue, which can consist of bismuth oxides and/or bismuth selenides. Moreover, it may form biaryls (Ar–Ar) as byproducts, but no biaryl was observed after the reaction.

## Conclusion

We have found that the photoinduced reaction of diaryl diselenides with triarylbismuthines affords unsymmetrical diaryl selenides in good yields. This method is efficient, because two arylseleno groups from diaryl selenides can be used as a selenium source, and its advantage is that the reaction proceeds in the absence of transition-metal catalysts.

## Experimental

### General comments

Compounds **1a**, **2a**, **3aa**, **4**, and **5** were obtained from commercially available materials. Diaryl diselenides **1b**–**e** [67] and triarylbismuthines **2b**–**d** [68] were synthesized according to the literature procedures.

### General procedure for the photoinduced synthesis of unsymmetrical diaryl selenides from diaryl diselenide and triarylbismuthine

(Ar^1^Se)_2_ (0.1 mmol), and Ar^2^_3_Bi (0.3 mmol) were dispersed in CH_3_CN (2 mL) with a stirring bar in a quartz test tube (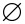 = 9 mm) with a septum in which a needle was inserted. The mixture was stirred and irradiated by a xenon lamp for 5 h at room temperature. The reaction mixture was filtered through a bed of celite (Celite 535). The crude product was purified by preparative TLC on silica gel (eluent: hexane/EtOAc). Details about compounds **3ab** [30], **3ac** [14], **3ad** [17], **3ba** [17], **3bb** [31], **3ca** [17], **3da** [32], **3ea** [28] and **3dc** [30] were reported in the corresponding articles.

## Supporting Information

File 1 Spectral and analytical data of the new compound **3cb**.
