# Supplementary material for: Photoinduced synthesis of unsymmetrical diaryl selenides from triarylbismuthines and diaryl diselenides
Source: Beilstein J Org Chem. 2013 Jun 13;9:1141–7. doi: 10.3762/bjoc.9.127 (PMC3701414; doi:10.3762/bjoc.9.127)
Supplement: File 1 — Spectral and analytical data of the new compound 3cb. [file Beilstein_J_Org_Chem-09-1141-s001.pdf]

Supporting Information

for

Photoinduced synthesis of unsymmetrical diaryl selenides from triarylbismuthines and diaryl diselenides

Yohsuke Kobiki, Shin-ichi Kawaguchi, Takashi Ohe and Akiya Ogawa*

Address: Department of Applied Chemistry, Graduate School of Engineering, Osaka Prefecture University, 1-1 Gakuen-cho, Nakaku, Sakai, Osaka 599-8531, Japan

Email: Akiya Ogawa - ogawa@chem.osakafu-u.ac.jp

* Corresponding author

Spectral and analytical data of the new compound 3cb

4-Tolyl 4-(trifluoromethyl)phenyl selenide (3cb): white solid; mp 104–105 °C; ^{1}H NMR (400 MHz, $CDCl_3$) δ 2.37 (s, 3H), 7.17 (d, *J* = 7.8 Hz, 2H), 7.36 (d, *J* = 8.2 Hz, 2H), 7.42 (d, *J* = 8.2 Hz, 2H), 7.48 (d, *J* = 7.8 Hz, 2H); ^{13}C NMR (100 MHz, $CDCl_3$) δ 21.2, 124.1 (q, $J_{C\text{-}F}$ = 271.7 Hz), 124.5, 125.5 (q, $J_{C\text{-}F}$ = 3.9 Hz), 128.4 (q, $J_{C\text{-}F}$ = 32.6 Hz), 130.2, 130.6, 135.4, 138.7, 139.0; ^{19}F NMR (376 MHz, $CDCl_3$) δ −62.4; ^{77}Se NMR (75 MHz, $CDCl_3$) δ 418; HRMS–FAB (*m*/*z*): $[M + H]^+$ calcd for $C_{14}H_{12}F_3Se$: 317.0056; found, 317.0077.

NMR Spectra

Copies of ^{1}H NMR, ^{13}C NMR, ^{19}F NMR, ^{77}Se NMR spectra of compound **3cb**.

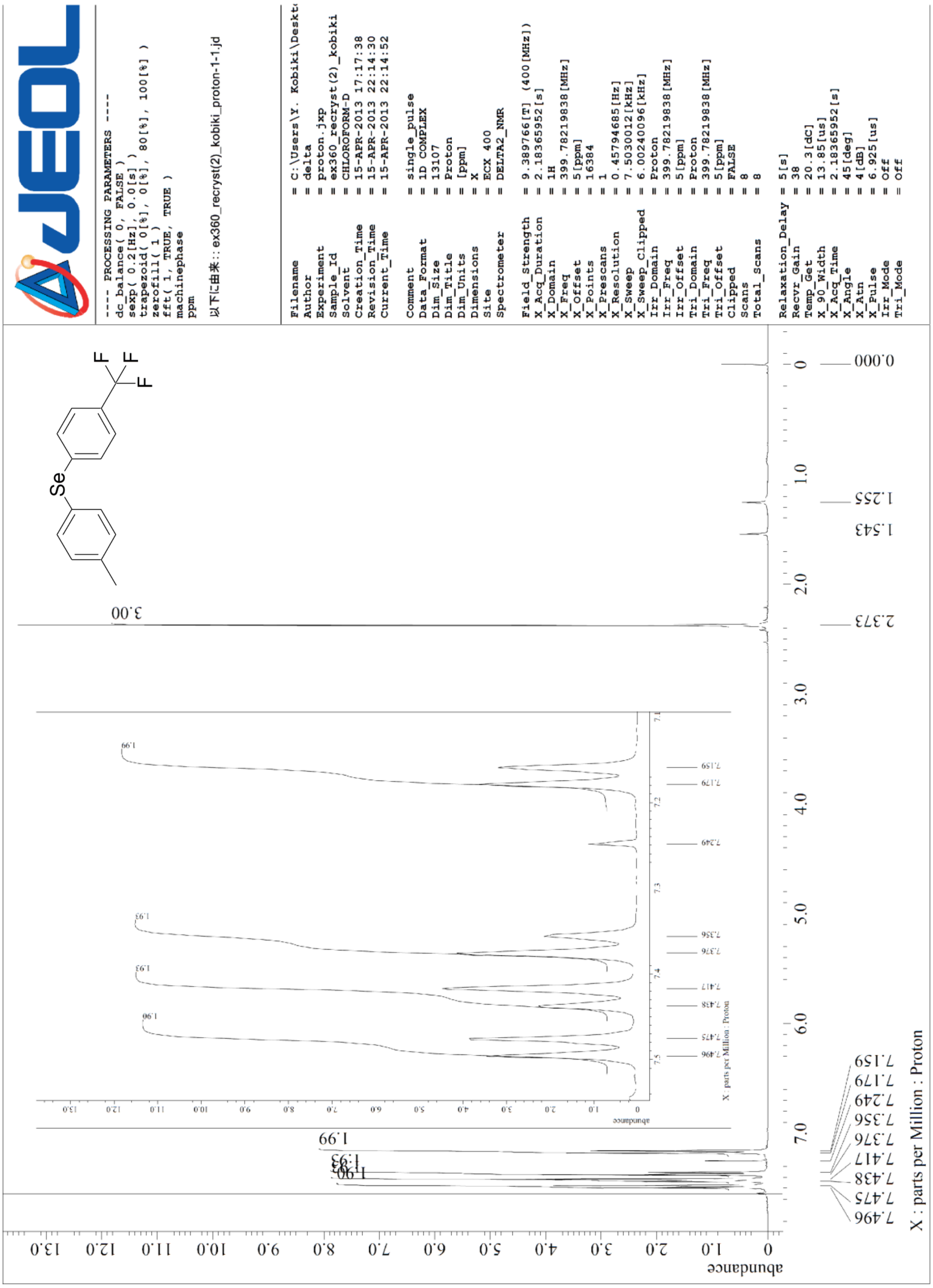

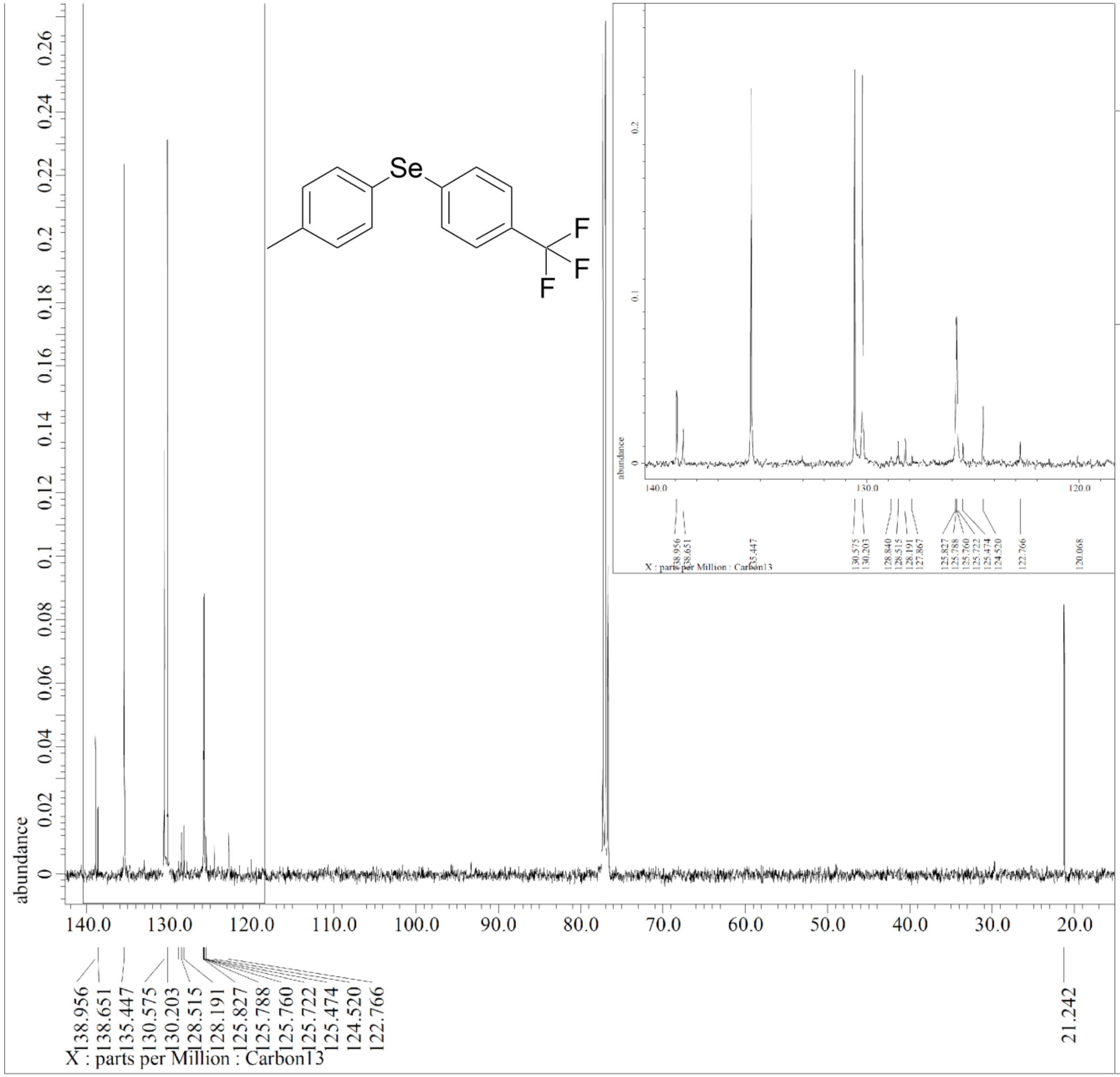


```
---- PROCESSING PARAMETERS ----
dc_balance( 0, FALSE )
sexp( 2.0[Hz], 0.0[s] )
trapezoid( 0[%], 0[%], 80[%], 100[%] )
zerofill( 1 )
fft( 1, TRUE, TRUE )
machinephase
ppm

以下に由来:: ex360_recryst(2)_kobiki_Carbon-1-1.jd

Filename          = C:\Users\Y. Kobiki\Deskt
Author            = delta
Experiment        = carbon.jxp
Sample_Id         = ex360_recryst(2)_kobiki
Solvent           = CHLOROFORM-D
Creation_Time     = 15-APR-2013 17:05:01
Revision_Time     = 15-APR-2013 22:20:18
Current_Time      = 15-APR-2013 22:20:55

Comment           = single pulse decoupled g
Data_Format       = 1D COMPLEX
Dim_Size          = 26214
Dim_Title         = Carbon13
Dim_Units         = [ppm]
Dimensions        = X
Site              = ECX 400
Spectrometer      = DELTA2_NMR

Field_Strength    = 9.389766[T] (400[MHz])
X_Acq_Duration    = 1.04333312[s]
X_Domain          = 13C
X_Freq            = 100.52530333[MHz]
X_Offset          = 100[ppm]
X_Points          = 32768
X_Prescans        = 4
X_Resolution      = 0.95846665[Hz]
X_Sweep           = 31.40703518[kHz]
X_Sweep_Clipped   = 25.12562814[kHz]
Irr_Domain        = Proton
Irr_Freq          = 399.78219838[MHz]
Irr_Offset        = 5[ppm]
Clipped           = TRUE
Scans             = 226.0
Total_Scans       = 226.0

Relaxation_Delay  = 2[s]
Recvr_Gain        = 46
Temp_Get          = 20.7[dC]
X_90_Width        = 9.92[us]
X_Acq_Time        = 1.04333312[s]
X_Angle           = 30[deg]
X_Atn             = 10[dB]
X_Pulse           = 3.30666667[us]
Irr_Atn_Dec       = 22.38[dB]
Irr_Atn_Noe       = 22.38[dB]
Irr_Noise         = WALTZ
Irr_Pwidth        = 0.115[ms]
Decoupling        = TRUE
```

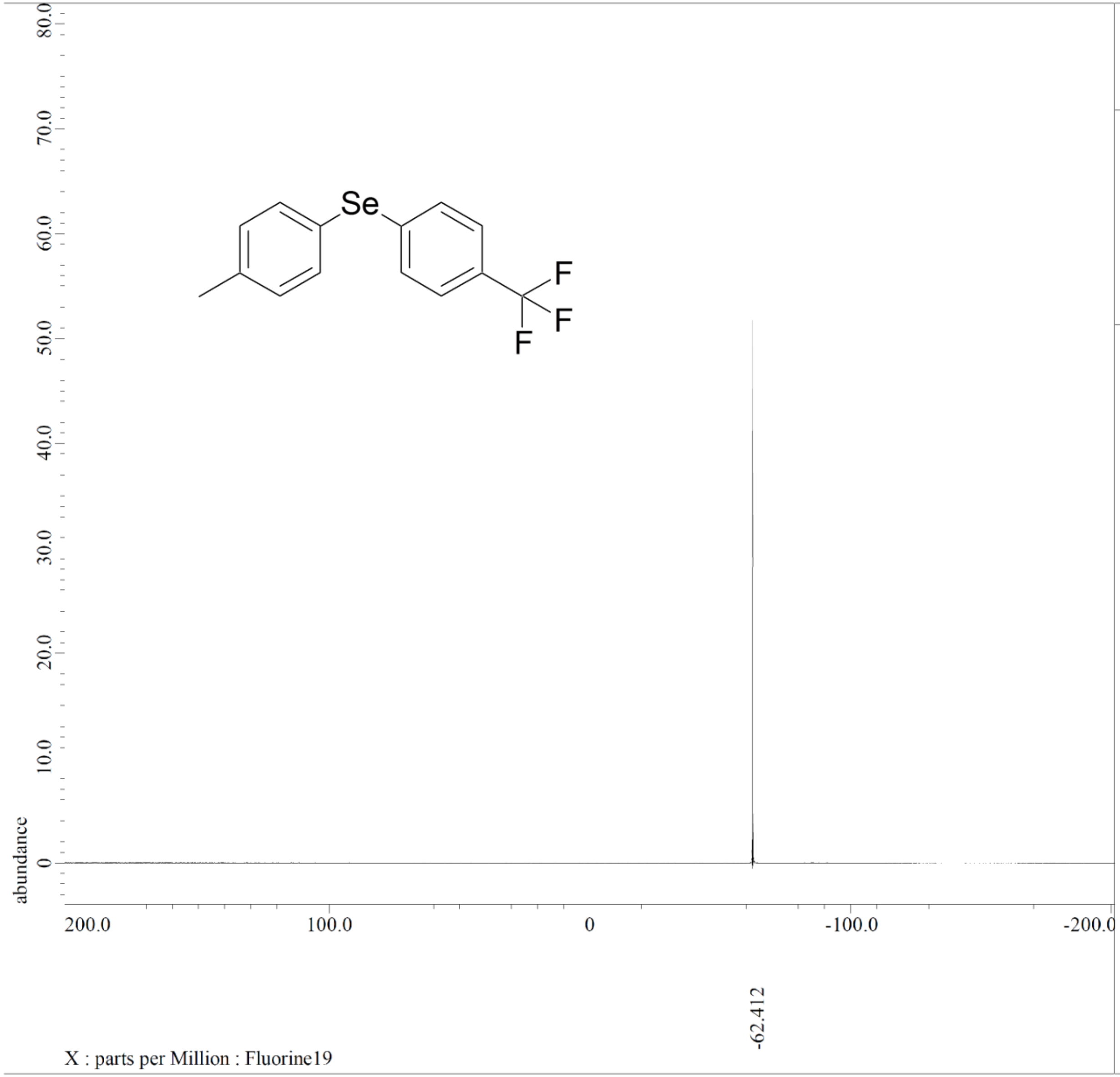


```
---- PROCESSING PARAMETERS ----
dc_balance( 0, FALSE )
sexp( 0.2[Hz], 0.0[s] )
trapezoid( 0[%], 0[%], 80[%], 100[%] )
zerofill( 1 )
fft( 1, TRUE, TRUE )
machinephase
ppm
```

以下に由来:: ex360_recryst_kobiki_Fluorine-1-1.jdf

```
Filename          = C:\Users\Y. Kobiki\Deskt
Author            = delta
Experiment        = proton.jxp
Sample_Id         = ex360_recryst_kobiki
Solvent           = CHLOROFORM-D
Creation_Time     =  9-APR-2013 17:12:39
Revision_Time     = 15-APR-2013 22:24:25
Current_Time      = 15-APR-2013 22:24:32

Comment           = single_pulse
Data_Format       = 1D COMPLEX
Dim_Size          = 13107
Dim_Title         = Fluorine19
Dim_Units         = [ppm]
Dimensions        = X
Site              = ECX 400
Spectrometer      = DELTA2_NMR

Field_Strength    = 9.389766[T] (400[MHz])
X_Acq_Duration    = 86.50752[ms]
X_Domain          = 19F
X_Freq            = 376.17105393[MHz]
X_Offset          = 0[ppm]
X_Points          = 16384
X_Prescans        = 1
X_Resolution      = 11.55968868[Hz]
X_Sweep           = 189.39393939[kHz]
X_Sweep_Clipped   = 151.51515152[kHz]
Irr_Domain        = Fluorine19
Irr_Freq          = 376.17105393[MHz]
Irr_Offset        = 5[ppm]
Tri_Domain        = Fluorine19
Tri_Freq          = 376.17105393[MHz]
Tri_Offset        = 5[ppm]
Clipped           = FALSE
Scans             = 32
Total_Scans       = 32

Relaxation_Delay  = 5[s]
Recvr_Gain        = 44
Temp_Get          = 20.6[dC]
X_90_Width        = 16.25[us]
X_Acq_Time        = 86.50752[ms]
X_Angle           = 45[deg]
X_Atn             = 4[dB]
X_Pulse           = 8.125[us]
Irr_Mode          = Off
Tri_Mode          = Off
```

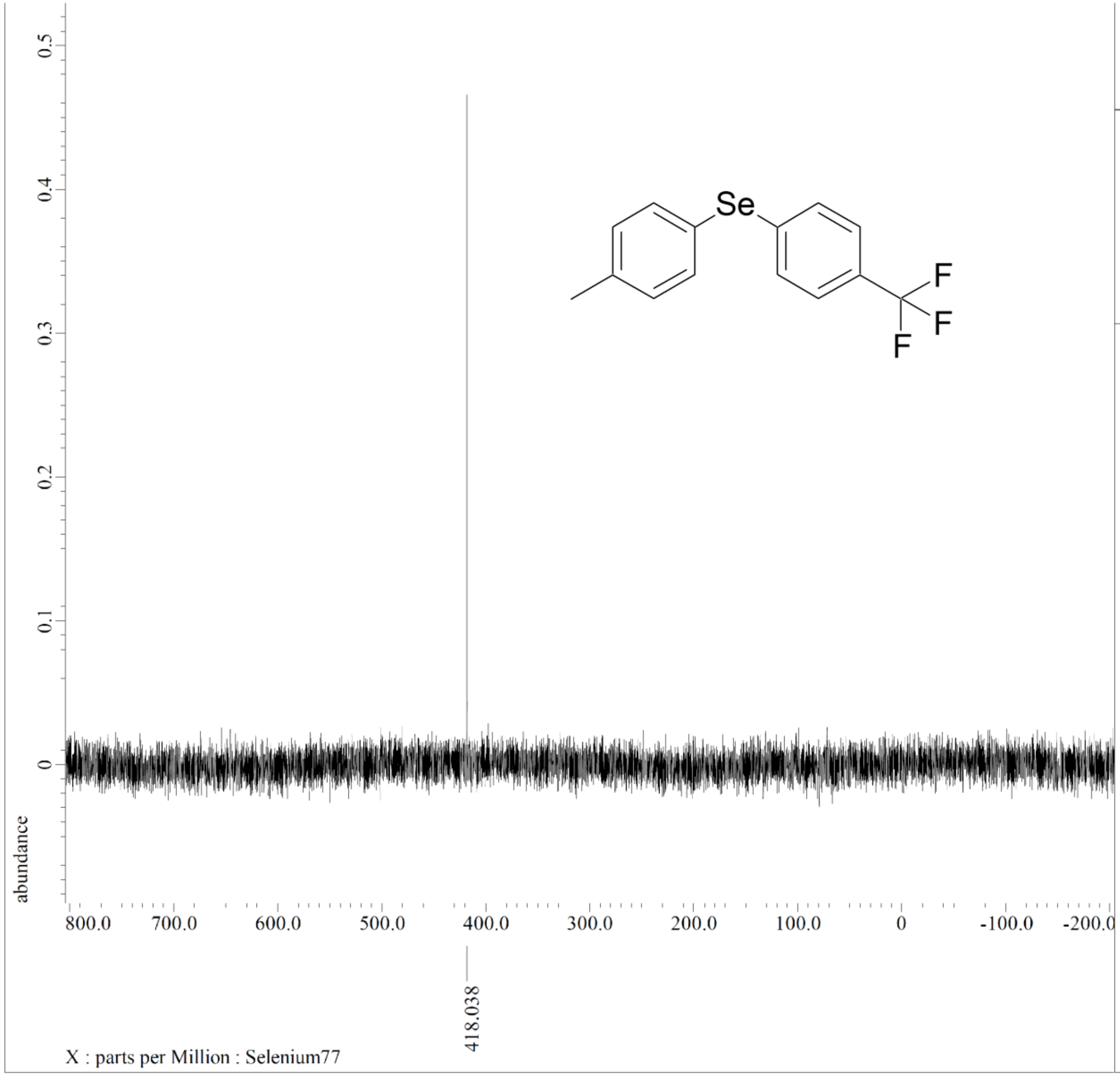


```
---- PROCESSING PARAMETERS ----
dc_balance( 0, FALSE )
sexp( 2.0[Hz], 0.0[s] )
trapezoid( 0[%], 0[%], 80[%], 100[%] )
zerofill( 1 )
fft( 1, TRUE, TRUE )
machinephase
ppm

以下に由来：: ex360_recryst_kobiki_Selenium-1-1.jdf

Filename            = C:\Users\Y. Kobiki\Deskt
Author              = delta
Experiment          = carbon.jxp
Sample_Id           = ex360_recryst_kobiki
Solvent             = CHLOROFORM-D
Creation_Time       =  9-APR-2013 17:21:31
Revision_Time       = 15-APR-2013 22:28:24
Current_Time        = 15-APR-2013 22:28:30

Comment             = single pulse decoupled g
Data_Format         = 1D COMPLEX
Dim_Size            = 26214
Dim_Title           = Selenium77
Dim_Units           = [ppm]
Dimensions          = X
Site                = ECX 400
Spectrometer        = DELTA2_NMR

Field_Strength      = 9.389766[T] (400[MHz])
X_Acq_Duration      = 0.3407872[s]
X_Domain            = 77Se
X_Freq              = 76.24442292[MHz]
X_Offset            = 300[ppm]
X_Points            = 32768
X_Prescans          = 4
X_Resolution        = 2.93438251[Hz]
X_Sweep             = 96.15384615[kHz]
X_Sweep_Clipped     = 76.92307692[kHz]
Irr_Domain          = Proton
Irr_Freq            = 399.78219838[MHz]
Irr_Offset          = 5[ppm]
Clipped             = TRUE
Scans               = 256
Total_Scans         = 256

Relaxation_Delay    = 2[s]
Recvr_Gain          = 56
Temp_Get            = 20.4[dC]
X_90_Width          = 10[us]
X_Acq_Time          = 0.3407872[s]
X_Angle             = 30[deg]
X_Atn               = 9[dB]
X_Pulse             = 3.33333333[us]
Irr_Atn_Dec         = 22.38[dB]
Irr_Atn_Noe         = 22.38[dB]
Irr_Noise           = WALTZ
Irr_Pwidth          = 0.115[ms]
Decoupling          = TRUE
```
